# Plate Load Tests on an Unsaturated Sand–Kaolin Mixture with Varying Water Table

**DOI:** 10.3390/s22062161

**Published:** 2022-03-10

**Authors:** Yi Tang, Chenghao Chen, Bin Qian, Jie Ren, Yunfei Guan

**Affiliations:** Department of Geotechnical Engineering, Nanjing Hydraulic Research Institute, Nanjing 210029, China; ytang@nhri.cn (Y.T.); chchen@nhri.cn (C.C.); bqian@nhri.cn (B.Q.); jren@nhri.cn (J.R.)

**Keywords:** plate load test, bearing capacity, unsaturated sand–kaolin mixture, water table, hydraulic hysteresis

## Abstract

Clayey sand is widely distributed and commonly encountered in geotechnical engineering practice. To understand its bearing capacity behavior under unsaturated conditions, plate load tests are performed on sand–kaolin mixture samples with varying water tables. The distributions of suction and volumetric water content with depth are measured by vibrating wire piezometers and soil moisture sensors, respectively. It is shown by the test results that the bearing capacity increases when the water table in the soil sample drops. The influence of suction on the bearing capacity is found to be dependent on the height of the water table and the hydraulic loading history of the soil sample. The plate load test results are interpreted using bearing capacity equations. Good agreement is obtained between measured and calculated bearing capacities. This study provides a simple method to estimate the bearing capacity of in situ unsaturated soil foundations.

## 1. Introduction

The plate load test (PLT) is conventionally used in engineering practice to measure the bearing capacity of foundations or back-calculate the soil parameters. Many in situ PLTs are performed on the surface of unsaturated soils since the compacted soil, earth structures, and roadways are commonly positioned above the groundwater table. Suction, which inherently exists in unsaturated soil, increases the bearing capacity by reinforcing the soil strength [1,2,3,4,5]. Therefore, the influences of suction should be accounted for in the interpretation of the PLT results.

Recently, PLTs have been performed on unsaturated clay and sand samples [2,6,7,8]. It is shown by the test data that suction has a significant influence on the bearing capacity. Clayey sand is a natural sedimentary soil widely distributed in estuaries and offshore areas. It has been shown that the mechanical and hydraulic behaviors of unsaturated clayey sand are different from those of clay and sand [9,10,11,12,13]. Few studies have been carried out to investigate the way of accounting for the influence of suction in the interpretation of PLT results of unsaturated clayey sand.

The seasonal fluctuation of the groundwater table due to infiltration and evaporation results in the changes in suction profile in the soil [14,15]. This natural phenomenon may also have significant influences on the bearing capacity. Research shows that the bearing capacity of unsaturated soil depends on the wetting or drying processes the soil has undergone even when the water table remains the same [3]. This is because the distribution of suction (*s*) and degree of saturation (*S*_r_) in the soil foundation are dependent on the hydraulic loading history due to hysteresis. However, few academic concerns have been spotlighted on how the changes of the water table could be taken into account when calculating the bearing capacity of unsaturated clayey sand.

In this paper, new PLTs were performed on samples of unsaturated sand–kaolin mixture. The water table in the sample is raised and lowered to simulate in-field hydrogeological conditions. The profiles of *s* and *S*_r_ are measured by vibrating wire piezometers and soil moisture sensors placed at different depths, respectively. Soil samples with a certain density were prepared to systematically investigate the influences of hydraulic loading history and water table on the PLT results. The results of the PLTs are interpreted using bearing capacity equations.

## 2. Test Soil

**Figure 1:**
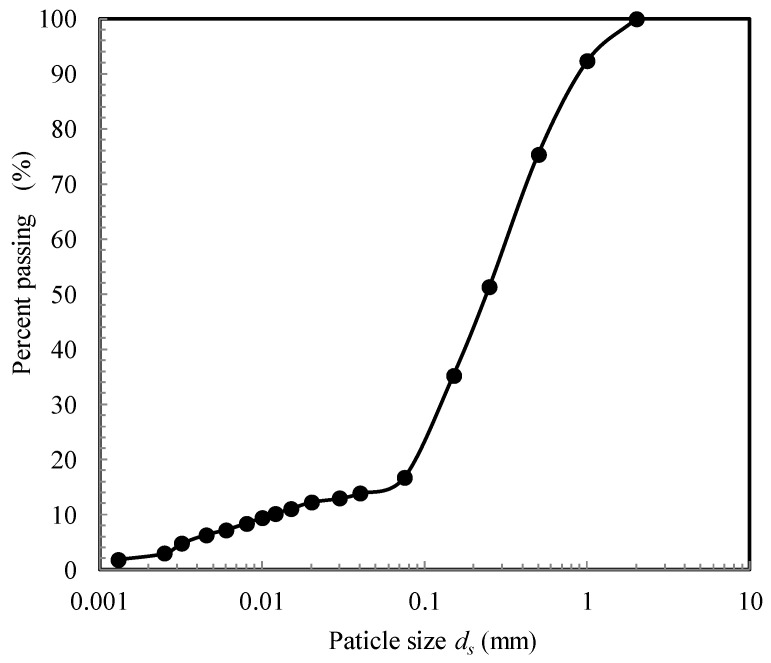
Particle size distribution of tested sand–kaolin mixture.

The test soil is made by mixing kaolin and clean sand with a respective proportion of 1/5 in dry weight. The specific gravity of the soil mixture is 2.66. The maximum and minimum dry densities (according to ASTM D4253-16 and ASTM D4254-16) [16,17] are 1.93 g/cm^3^ and 1.59 g/cm^3^, respectively. Figure 1 shows the grain size distribution curve. The friction angle (*φ*_cs_′) at the critical state is 34.8°.

**Figure 2:**
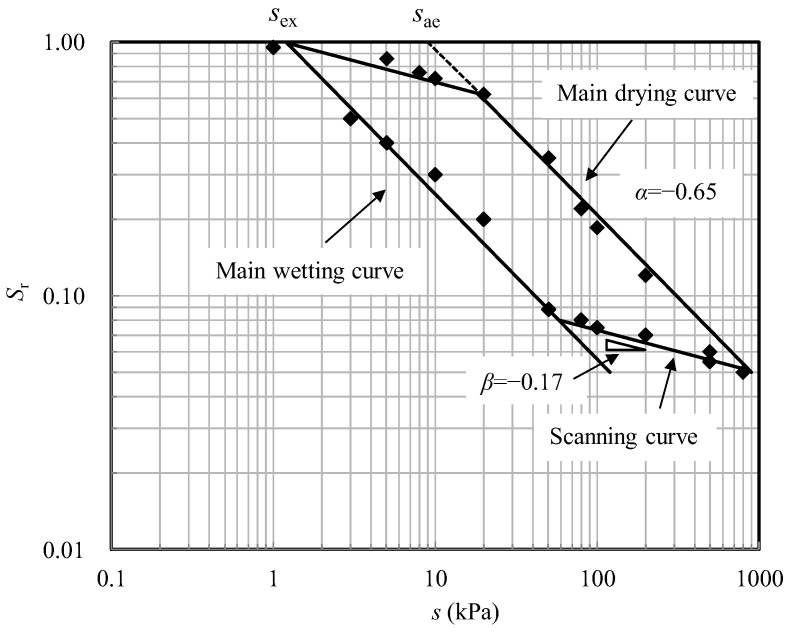
SWCC of sand–kaolin mixture with a void ratio 0.42.

The soil–water characteristic curve (SWCC) of the test soil is determined by plate load tests for samples with a void ratio of 0.42 (relative density of 0.85). The SWCC was plotted in double logarithmic scale (Figure 2) and can be fitted by the Russell model [18,19], which is defined as:

(1) $$S_{r}=\{\begin{array}{ccc} 1 & for & \frac{s}{s_{e}}\leq 1\\ {\left(\frac{s}{s_{e}}\right)}^{\alpha } & for\;main\;drying\;or\;wetting\;curves & \frac{s}{s_{e}}>1 \end{array}$$

(2) $$S_{r}=\{\begin{array}{c} {\left(\frac{s_{\mathrm{rd}}}{s_{\mathrm{ae}}}\right)}^{\alpha }{\left(\frac{s}{s_{\mathrm{rd}}}\right)}^{\beta }for\;drying\;path\;reversal\;\\ \;{\left(\frac{s_{\mathrm{rw}}}{s_{\mathrm{ex}}}\right)}^{\alpha }{\left(\frac{s}{s_{\mathrm{rw}}}\right)}^{\beta }for\;wetting\;path\;reversal\; \end{array}$$

where *s*_e_ is the threshold suction value distinguishing saturated and unsaturated states. *s*_ae_ and *s*_ex_ are the air-entry and air-expulsion values, respectively. *s*_rd_ and *s*_rw_ are the points of suction reversal on the main drying and main wetting paths, respectively. *α* = −0.65 is the slope of the main drying and main wetting curves. *β* = −0.17 is the slope of the scanning curves.

## 3. Plate Load Test

### 3.1. Test Apparatus

**Figure 3:**
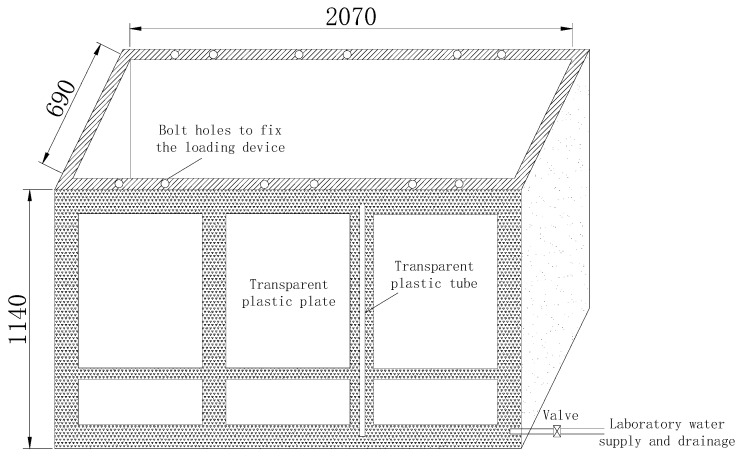
Diagrammatic sketch of the testing rig (in mm).

The plate load test apparatus is comprised mainly of a testing rig (shown in Figure 3), a loading system and a set of data acquisition devices. The size of the testing rig is 2.07 m × 0.69 m × 1.14 m (long × wide × deep). When preparing the soil samples, the height of the soil sample and water table can be monitored through the transparent plastic plate and the transparent plastic tube, respectively. Water is added and drained through a valve to raise and lower the water table to the target height. Bolt holes are drilled at the top edge of the testing rig to fix the loading devices at three different locations.

**Figure 4:**
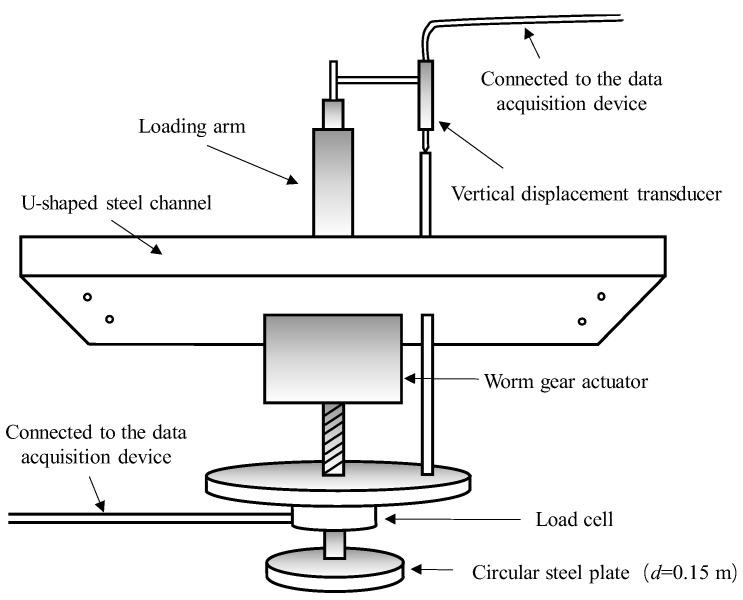
Diagrammatic sketch of the loading system.

A detailed design of the loading system is shown in Figure 4. A rigid steel plate, with a diameter equal to 150 mm, is utilized. The vertical displacement is applied by the worm gear actuator and measured by the vertical displacement transducer. The load is measured by the load cell which has a capacity of 45 kN. The loading system is connected to the data acquisition devices.

### 3.2. Sample Preparation

Two uniform and dense soil samples with an ultimate thickness of 480 mm and a relative density of 0.85 were made in three layers. The weight of soil in each layer was calculated to ensure the uniformity of the sample. The dry soil samples were placed on top of the geotextile above a 200 mm-thick gravel layer. The method of pluviation with a drop height of 50 cm was used [3,20]. A thin layer of sand with a different color was placed between two soil layers to guarantee the accurate measurement of the ultimate thickness of each soil layer. Then, the entire testing rig was vibrated with a frequency of 80 Hz. To prepare dense samples, preliminary trial tests were carried out, so that an adequate time of vibration can be determined. After vibration, the thickness of each layer was measured and found to be very close to 160 mm, indicating the uniformity of soil samples.

Then, these two samples were made unsaturated by subjecting them to different hydraulic processes: one subjected to wetting and the other one subjected to drying. Three PLTs were performed on each soil sample under saturated conditions and unsaturated conditions with different heights of water table.

To prepare the sample subjected to a wetting process, the level of the water table was maintained at the soil–gravel interface. Water moves upward due to capillary stress. The sample is assumed to fully reach the equilibrium state when the suction variations at different depths are less than 0.1 kPa during 24 h. Then, the water table was raised to 240 mm above the soil–gravel interface. The equilibrium states should have also been reached before the PLTs were carried out.

The samples were saturated by raising the water table to 20 mm above the soil surface. The fully saturated state was deemed to have been reached when all degrees of saturation measurements were larger than 0.95.

To prepare the sample subjected to a drying process, the water table in the saturated sample was lowered to the middle of the sample (240 mm above the geotextile) and the soil–gravel interface, respectively. The changes of suction during 24 h were less than 0.1 kPa before the PLTs were carried out under these two conditions.

**Figure 5:**
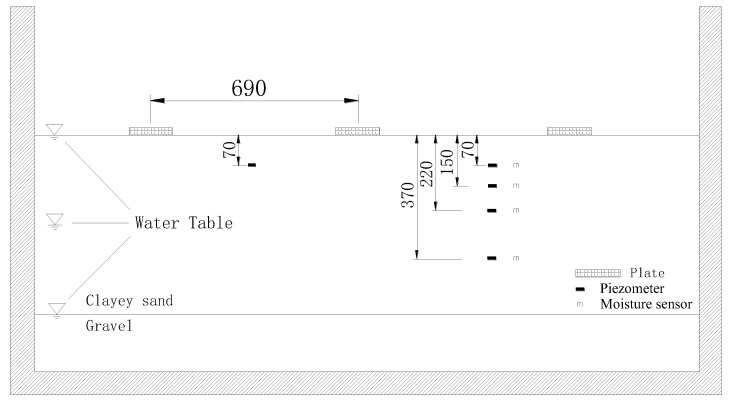
Different depths of water table and the arrangement of sensors.

In this study, the suction profiles in the soil sample were measured by vibrating wire piezometers due to their accurate and reliable measurements [21]. The piezometers with an accuracy of ±0.1% were positioned at the depths of 70 mm, 150 mm, 220 mm, and 370 mm (shown in Figure 5). The values of suction at the depth of 70 mm were measured by two piezometers installed at different locations. They ensure the suction measurements at shallow depth are reliable.

Theta-probe soil moisture sensors were used to measure the variation of volumetric water content with depth due to their high accuracy [22]. Four moisture sensors were inserted at the same depths of the piezometers, enabling the calculation of the degree of saturation corresponding to the measured suction and the determination of hydraulic states on the SWCC.

### 3.3. Test Program

To study the effect of water tables on the bearing capacity, three PLTs were performed on the surface of each soil sample: one test under fully saturated conditions, the other two tests under unsaturated conditions that the water table was at the same level of the geotextile and 240 mm higher than the geotextile, respectively. The distance between the location of each PLT is more than four times the plate diameter and is sufficient to avoid the interaction between two adjacent tests.

During the test procedure, the plate was loaded by applying a vertical displacement with a constant rate of 0.02 mm/s. The tests were terminated when the measured load stopped increasing, and a further displacement of about 10 mm was applied. A PLT lasts for 30 min on average, during which the pivotal information with respect to the load and the vertical displacement was continuously recorded.

## 4. Plate Load Test Results

**Figure 6:**
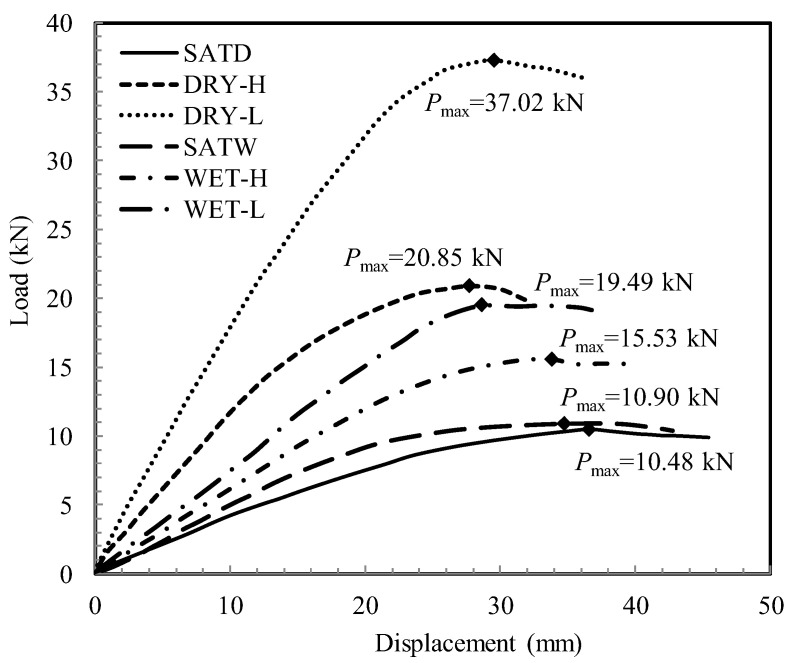
Plate load test results.

Figure 6 displays the load–displacement curves obtained from the PLTs. The information of the PLTs and the bearing capacities calculated by dividing the peak loads by the area of the plate are listed in Table 1. Table 2 provides the values of suction and degree of saturation measured at multiple depths. The identity of two suction measurements at the depth of 70 mm verifies the uniformity of the soil sample.

It is shown in Figure 6 that during the tests, the load increases with the applied displacement to a peak value before a slight drop or fluctuation. For a particular sample, the peak loads for unsaturated conditions are significantly larger than those for saturated conditions. Almost identical load–displacement curves are obtained for two samples under saturated conditions, indicating that the soil samples have a similar density and the test results are reliable.

## 5. Interpretation of PLT Results Considering Influences of Suction and Water Table

### 5.1. Influence of Suction on the Bearing Capacity

As can be seen in Table 1 and Table 2, suction develops in unsaturated samples and increases the bearing capacity. In comparison with the saturated conditions, the bearing capacity is increased by about 2 times and 3.5 times for the soil sample subjected to drying, and about 1.4 times and 1.8 times for the soil sample subjected to wetting. For the soil sample subjected to drying, the most striking increase in the bearing capacity is obtained when the water table is at the soil–gravel interface. This is because the values of suction developed within the soil sample are the largest.

### 5.2. Effect of Water Table on the Bearing Capacity

It is seen in Figure 6 that the water table has significant influences on the bearing capacity. For an unsaturated sample having undergone a certain hydraulic process (drying or wetting), the bearing capacity becomes larger with a descent of the water table. The difference can be attributed to the different suction profiles developed in the sample, as shown in Table 2.

### 5.3. Effect of Hydraulic Loading History on the Bearing Capacity

For samples subjected to different hydraulic processes, the measured bearing capacities are different regardless of the same level of the water table. The bearing capacity is intensely related to the hydraulic loading history the sample has experienced. For instance, it can be found that the bearing capacity measured by the test DRY-H is even larger than that measured by the test WET-L.

The impact of suction on the effective stress and bearing capacity could be accounted for by *χs*, where *χ* is the effective stress parameter [14,23]. *χ* can be calculated following the expressions proposed in [24], which consider the influence of hydraulic hysteresis:

(3) $$\chi =\{\begin{array}{ccc} 1 & for & \frac{s}{s_{e}}\leq 1\\ {\left(\frac{s}{s_{e}}\right)}^{\Omega } & for\;main\;drying\;or\;wetting\;curves & \frac{s}{s_{e}}>1 \end{array}$$

(4) $$\chi =\{\begin{array}{c} {\left(\frac{s_{\mathrm{rd}}}{s_{\mathrm{ae}}}\right)}^{\Omega }{\left(\frac{s}{s_{\mathrm{rd}}}\right)}^{\zeta }for\;drying\;path\;reversal\;\\ \;{\left(\frac{s_{\mathrm{rw}}}{s_{\mathrm{ex}}}\right)}^{\Omega }{\left(\frac{s}{s_{\mathrm{rw}}}\right)}^{\zeta }for\;wetting\;path\;reversal\; \end{array}$$

where *Ω* is a material parameter that has a best-fit value of −0.55. *ζ* = *βΩ*/*α* = −0.14 is the slope of the scanning curves in ln*χ*~ln*s* plane.

Based on these expressions, the value of *χ* is determined by suction and the location of the hydraulic state on the SWCC. During the sample preparation, the water table in the soil changes under different hydraulic processes to reach the target height. For the sample undergoing drying, the hydraulic state starts from the fully saturated condition and moves down along the top scanning curve. Then, it moves onto the main drying curve when the water table is continuously lowered down and the corresponding suction exceeds a certain value. For the sample subjected to wetting, the moisture content increases and the hydraulic state moves up, following the main wetting curve.

**Figure 7:**
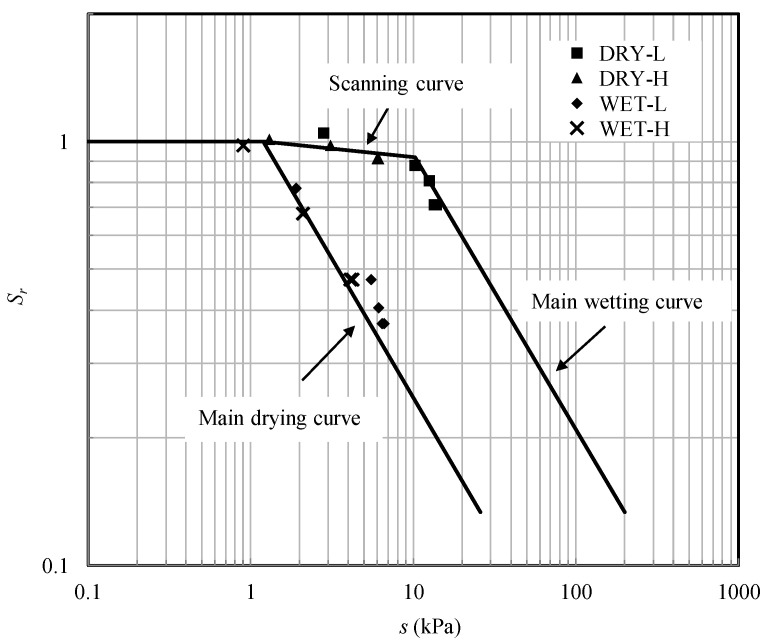
Illustration of the hydraulic states locations on the SWCC.

Figure 7 plots the measured values of suction and degree of saturation at different depths on the SWCC prior to testing. It can be seen that the hydraulic states for the sample undergoing drying are on the top scanning curve and the main drying curve while those for the sample undergoing wetting are on the main wetting curve. As shown in Table 2, the values of *χs* for the test DRY-H is larger than those for the tests WET-L, and thus, a larger bearing capacity was measured.

### 5.4. Interpretation Using Bearing Capacity Equations

**Figure 8:**
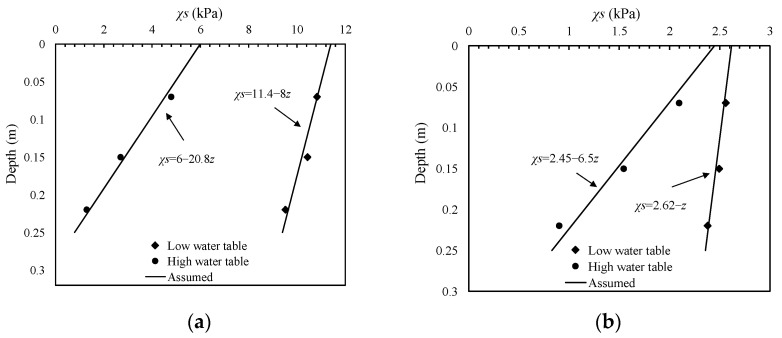
Assumed *χs* profiles for unsaturated soil samples subjected to (**a**) drying; (**b**) wetting.

The values of *χs* decrease with depth. It has been found that the stress and suction within the depth of 1.5 *B* (*B* is the footing diameter) in the soil have more profound influences on the bearing capacity [2,3,25]. Therefore, linear profiles fitting the measured values well within the shallow parts of the samples could be assumed to quantitatively evaluate the influence of *χs* on the bearing capacity [3]. Figure 8 shows these linear *χs* profiles.

For the sample undergoing drying, the *χs* profile is assumed as *χs* = 11.4 − 8*z* when the water table is at the soil–gravel interface and *χs* = 6 − 20.8*z* when the water table is at a height of 240 mm.

For the sample undergoing wetting, the *χs* profile is assumed as *χs* = 2.62 − *z* when the water table is at the soil–gravel interface and *χs* = 2.45 − 6.5*z* when the water table is at a height of 240 mm.

According to the effective stress principle and the slip line theory, the effects of linear *χs* profile can be incorporated into the bearing capacity equation similar to the linear varied *c*′ in saturated soil [3,14]. An equation for calculation of the bearing capacity (*q*_u_) of footing on the surface of unsaturated soil can be derived as:

(5) $$q_{u}=\left(c^{\prime }+{\left(\chi s\right)}_{0}\tan\varphi^{\prime }\right)N_{c}+\frac{1}{2}\left(K_{\chi s}+\gamma_{t}\right)BN_{\gamma }$$

where (*χs*)_0_ is the *χs* value at the soil surface, *K_χs_* is a constant representing the changes in *χs* with respect to depth, *γ*_t_ is the soil total unit weight, *N*_c_ and *N*_γ_ are the bearing capacity factors related to the soil friction angle.

Equation (5) is in agreement with Terzaghi’s bearing capacity equation when the soil is dry or fully saturated. The values of *q*_u_ are calculated using Equation (5) for different conditions and listed in Table 1. A peak friction angle of *φ*′_peak_ = 37° is used in the calculation. This value can be verified by the results of PLTs performed on saturated samples. Values of bearing capacity factors for rough circular footing (*N*_c_ = 147.9, *N*_γ_ = 63.4) are used [26].

**Figure 9:**
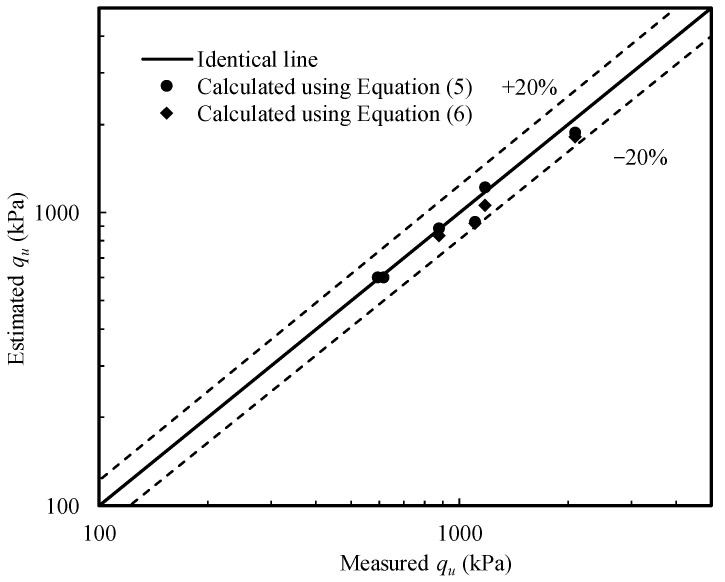
Comparison between measured and calculated bearing capacities.

The *q*_u_ values calculated using Equation (5) are shown in Figure 9 and compared with the measured bearing capacities under variably saturated conditions. It can be seen that a reasonable good agreement is obtained and the associated errors are less than 20% for all cases.

The assumed linear *χs* profile within the depth of 1.5 *B* could be used in the bearing capacity calculation with a good accuracy. The value of *χs* at the depth of 0.75 *B*, (*χs*)_mid_, which is the average value of *χs* for this profile, may be taken as the representative value to consider the effect of suction. Therefore, Equation (5) is simplified to be:

(6) $$q_{u}=\left(c^{\prime }+{\left(\chi s\right)}_{\mathrm{mid}}\tan\varphi^{\prime }\right)N_{c}+\frac{1}{2}\gamma_{t}BN_{\gamma }$$

The values of (*χs*)_mid_ are derived from the assumed linear *χs* profiles. Figure 9 shows the calculated *q*_u_ values that match the measured values well.

For most cases in engineering practice, the footings are built on the surface of the unsaturated soil layer above the ground water table. Therefore, it is important to evaluate the effect of suction on the bearing capacity for a more accurate and realistic foundation design. If the water table is lower than 1.5 *B* below the footing, the bearing capacity may be simply estimated using the measurements of one vibrating wire piezometer and one soil moisture sensor installed at the depth of 0.75 *B*.

## 6. Conclusions

In this paper, PLTs are performed on a sand–kaolin mixture under varying water table conditions. Two uniform saturated and unsaturated samples with a relative density of 0.85 were prepared. Wetting and drying processes of soil samples were replicated by raising and lowering the water table, respectively. The vibrating wire piezometers disposed at different depths provide measurements of suction profiles in unsaturated samples. The soil moisture sensors were installed at the corresponding depths to measure the volumetric water content. The variations in *χs* with depth are determined by the measurements of suction and the locations of the hydraulic state on the SWCC.

Test results show that the bearing capacity of the sand–kaolin mixture is significantly influenced by the water table and the hydraulic loading history it has experienced. Bearing capacity equations derived from the slip line theory are applied to interpret the PLT results. The linear *χs* profiles within the shallow part of the soil sample that affect the bearing capacity to a great extent and the representative *χs* value at the depth of 0.75 *B* are used in the calculation. The computed values agree reasonably well with the PLT data and the differences for all cases are less than 20%.

The findings of this study may provide a convenient method to estimate the bearing capacity of unsaturated soil foundations with reasonable accuracy. The only required measurements are the SWCC of the foundation soil, suction, and volumetric water content at the depth of 0.75 *B*. The applicability and reliability of this method could be evaluated by further laboratory and in situ tests performed on different types of unsaturated soils.

## Figures and Tables

**Table 1:** Measured and estimated bearing capacities for PLTS under different conditions.

Test	Condition	Water Table	Measured *q*_u_ (kPa)	Calculated *q*_u_ Using Equation (5) (kPa)
SATD	Saturated	Soil surface	593	602
DRY-H	Unsaturated drying	At a height of 240 mm	1180	1218
DRY-L	Unsaturated drying	Soil-gravel interface	2095	1878
SATW	Saturated	Soil surface	617	602
WET-H	Unsaturated wetting	At a height of 240 mm	879	885
WET-L	Unsaturated wetting	Soil-gravel interface	1103	927

**Table 2:** Index properties of the unsaturated samples at multiple depths.

Test	Depth (m)	Volumetric Water Content	*S* *r*	*s* (kPa)	Calculated *χs* (kPa)
DRY-H	0.07	0.27	0.91	6.0, 6.1	4.78
	0.15	0.29	0.98	3.1	2.70
	0.22	0.30	1	1.3	1.29
DRY-L	0.07	0.21	0.71	13.4, 13.8	10.83
	0.15	0.24	0.81	12.5	10.43
	0.22	0.26	0.88	10.2	9.52
	0.37	0.31	1	2.8	2.48
WET-H	0.07	0.14	0.47	4.1, 4.2	2.10
	0.15	0.20	0.68	2.1	1.54
	0.22	0.29	0.98	0.9	0.90
WET-L	0.07	0.11	0.37	6.4, 6.6	2.56
	0.15	0.12	0.41	6.1	2.49
	0.22	0.14	0.47	5.5	2.38
	0.37	0.23	0.78	1.9	1.48
